# Mental health support for British Bangladeshi youth: multi-stakeholder qualitative study of priorities and preferences

**DOI:** 10.1192/bjo.2026.10999

**Published:** 2026-03-10

**Authors:** Ishrat Shahnaz, Hannah Kate Lewis, Kelly Rose-Clarke, Daniel Michelson, Petra C. Gronholm, Erica Cini

**Affiliations:** Department of Child and Adolescent Psychiatry, Institute of Psychiatry, Psychology & Neuroscience, King’s College London, London, UK; Department of Psychology, University of Dhaka, Dhaka, Bangladesh; Centre for Psychiatry and Mental Health, Wolfson Institute of Population Health, Queen Mary University of London, London, UK; UCL Institute for Global Health, University College London, London, UK; NIHR Maudsley Biomedical Research Centre, South London and Maudsley NHS Foundation, London, UK; Centre for Global Mental Health, Department of Population Health, London School of Hygiene & Tropical Medicine, London, UK; Eating Disorder Intensive Programme, East London NHS Foundation Trust, London, London, UK; Nutrition Science Group, Division of Medicine, University College London, London, UK

**Keywords:** British Bangladeshi youth, mental health challenges, improving service access, community-led mental health services, culturally appropriate care

## Abstract

**Background:**

Despite young British Bangladeshis experiencing a higher prevalence of mental health problems than the White British population, they are comparatively underrepresented in mental health research and fewer access services.

**Aims:**

This study aimed to identify priorities and preferences for mental health support among young British Bangladeshis, as well as strategies to enhance the cultural appropriateness and accessibility of services.

**Method:**

A qualitative study was conducted in London and Leicester, with participants recruited through voluntary and community organisations. In-depth interviews were held with young people with lived experience of mental health problems (*n* = 12), their family members (*n* = 7) and Bangladeshi community workers from voluntary sector organisations (*n* = 7). Data were analysed using thematic framework analysis.

**Results:**

Participants’ priorities for mental health support included reducing stigma, raising awareness, and addressing intergenerational and identity-related stressors. Preferences centred on culturally and linguistically appropriate care delivered by therapists familiar with Bangladeshi values in trusted community settings. Suggested strategies for improving existing services included enhancing access by providing support in trusted community spaces, building trust through the involvement of culturally aware professionals, and involving families and community leaders to reduce stigma and promote open discussion around youth mental health.

**Conclusions:**

This study identifies a need for culturally tailored, linguistically accessible and community-rooted mental health support for British Bangladeshi youth. Representatives from these communities should be actively involved in the development of future programmes and policies, ensuring that support is both relevant and sustainable.

The UK has the largest Bangladeshi population outside Bangladesh and India. According to the 2021 Census, 652 535 Bangladeshis lived in the UK, representing just under 1% of the total population.^1^ This community is one of the youngest ethnic groups in the country, with a notably younger age profile than other South Asian communities.^2,3^ Evidence suggests that British Bangladeshi people experience higher rates of mental ill health than White British individuals, yet report lower rates of diagnosed mental illness than other ethnic groups.^2,4^ Although youth-specific prevalence data are limited, adult studies and indicators such as low service uptake and high exposure to psychosocial stressors point to substantial unmet need.^2^ Socioeconomic challenges such as low income, unemployment, overcrowded housing and racism further impact British Bangladeshis’ mental health.^5,6^

## Cultural conflict, identity stress and barriers to service use

Beyond structural inequalities, cultural dissonance between traditional Bangladeshi values at home and broader British society exacerbates these struggles, particularly for younger British Bangladeshis.^5^ This is especially critical for young people between the ages of 16 and 24 years, a developmental period characterised by rapid psychological, social and neurological changes, alongside increasing societal expectations around independence, education, employment and relationships.^7^ This is also the peak period for the onset of emotional and behavioural disorders, and the clash between home and societal norms at this stage can intensify identity conflict and distress, underlining the need for developmentally and culturally responsive mental health support.^8–10^ Despite these risks, British Bangladeshis have some of the lowest referral rates to talking therapies,^11,12^ possibly because of mistrust of services and reliance on informal or community-based support. Barriers include perceived discrimination from healthcare providers, lack of cultural sensitivity, and limited awareness and trust in mental health services.^5^ Stigma and distrust in mental health services remain significant barriers to accessing support, particularly for Bangladeshi men aged 22–59 years.^13^

## Community-based support and the need for ethnicity-specific research

Community-based organisations offer an important complement to statutory mental health services.^14^ For example, the Bangladeshi Mental Health Forum in East London provides Bengali-language workshops, peer support groups, professional training, and partnerships with National Health Service (NHS) Trusts to improve service access and relevance.^15^ Camden Council has taken strategic steps to address health inequalities in the Bangladeshi community through awareness and engagement,^16^ and Roshni Ghar in West Yorkshire offers culturally sensitive support and safe spaces for South Asian women, including Bangladeshi, Indian and Pakistani communities, experiencing psychological distress.^17^ However, they remain limited in scale and are rarely tailored to the specific developmental and cultural needs of younger British Bangladeshis. Although a substantial body of research has explored mental health experiences and service access among South Asian communities in Britain, these studies often conceptualise South Asians as a single, homogeneous group, overlooking the sociocultural and historical differences among Indian, Pakistani, Bangladeshi and other subgroups. There is a need for more granular, ethnicity-specific investigations to better understand the distinctive strengths and challenges of these communities.^18^

## Current study

Building on this context, this qualitative study investigates the mental health needs of British Bangladeshi youth, focusing on the following research questions:

- How do British Bangladeshi youth (aged 16–24 years) describe and experience mental health challenges in the UK?
- What are the strengths and limitations of mental health support systems and services available for this target group?
- How could mental health support systems and services be developed to better account for the cultural, linguistic, socioeconomic and other factors that are specifically relevant to the lived experiences of young Bangladeshi people in the UK?
- What are the barriers and opportunities for involving community-based organisations and local leaders in mental health support for young people of Bangladeshi origin in the UK?

The study aims to generate in-depth, contextually grounded insights into the lived experiences of British Bangladeshi youth, rather than determine the prevalence or representativeness of mental health problems.

## Method

### Design and setting

This qualitative study used semi-structured interviews with British Bangladeshi youth, parents and community workers from third-sector organisations. Data were collected between February and August 2024 in London and Leicester, with participants recruited through mental health and youth-focused charities from the Bangladeshi community. Reporting follows the COREQ (Consolidated Criteria for Reporting Qualitative Research) checklist to ensure transparency and rigour (see Supplementary File 1).^19^ All procedures comply with the ethical standards of the national and institutional committees on human experimentation and with the Helsinki Declaration of 1975, as revised in 2013, and were approved by the Health Faculties Research Ethics Subcommittee at King’s College London (reference: HR/DP-23/24-40530).

### Eligibility

Eligible participants were Bangladeshi young people aged 16–24 years who had experienced stressful events affecting their well-being or had insight into peer’s or family member’s mental health challenges, along with their parents, and Bangladeshi community workers from community and voluntary sector organisations with shared interests in the mental health and well-being of Bangladeshi youth. Given the study’s aim to inform culturally and contextually appropriate early interventions, formal mental health screenings were not conducted. Instead, participants were included based on a perceived need for psychosocial support, regardless of diagnostic criteria. The final sample (*N* = 26) included 12 young people, seven parents (each corresponding to an index young person) and seven community workers. Young people were purposively and snowball sampled to ensure diversity in age, gender and location (London and Leicester). Parents reflected varied family experiences, whereas community workers were recruited based on their roles supporting British Bangladeshi youth. The sample size was determined by the information power principle^20^ and thematic saturation,^21^ which was achieved after 26 interviews, when no new themes or insights emerged from the data.

### Participant recruitment

Recruitment materials, including flyers, participant information sheets and consent forms, were translated into Bangla for parents and community workers who may not be fluent in English. Recruitment was conducted via online and social media platforms (e.g. university portals, WhatsApp groups), and through three collaborating organisations that distributed flyers in community spaces (e.g. libraries, town halls and supermarkets in localities with relatively large Bangladeshi communities). Interested individuals registered their interest via email or a scanned QR code, after which the first author (I.S.) contacted them. All participants received an information sheet and provided written informed consent before participation, and none withdrew. Participants received a £20 voucher and travel reimbursement.

### Data collection

Semi-structured interviews drew on a bilingual (English/Bengali) topic guide (see Supplementary File 2), including questions on mental health challenges, support needs and preferences of young Bangladeshi people. Interviews (45–60 min) were conducted online, by telephone or in person at collaborating organisations. I.S., a bilingual researcher, conducted, recorded and transcribed all interviews. Online interviews conducted via Microsoft Teams were auto-transcribed using its built-in feature, and then manually checked and corrected for accuracy. All other interviews were transcribed manually by the first author (I.S.). Bengali transcripts were translated into English by the first author (I.S.).

### Data analysis

Interviews were analysed using framework analysis^22^ involving transcription, familiarisation, thematic framework development, indexing, charting, and mapping and interpretation.^23^ Data were managed and coded using NVivo software, version 14 for macOS (Lumivero, Melbourne, Australia; https://lumivero.com/products/nvivo/). Both inductive and deductive coding were used. Deductive coding was informed by the research questions, topic guide and three theoretical frameworks: Bronfenbrenner’s ecological systems theory,^24,25^ Gask et al’s Process Model of Access ^26^ and the PLACES model.^27^ These frameworks supported analysis of environmental influences, help-seeking processes, and culturally accessible service design. Additionally, inductive data-driven coding was applied.

I.S. and H.K.L. independently coded four transcripts. An initial analytical framework was developed collaboratively and refined through discussion with senior co-authors (D.M., P.C.G. and E.C.). I.S. then applied the framework to the full data-set, charting data into a matrix to compare cases and identify patterns. Iterative refinement of codes led to the final themes and subthemes. As an academic psychologist, I.S.’s shared cultural background with participants facilitated rapport and contextual insight. However, differences in migration histories required careful reflexivity to avoid assumptions. Regular discussion with team members from diverse professional and cultural backgrounds ensured analytical rigour and interpretive balance.

## Results

### Participants

Participant characteristics are summarised in Table 1.

**Table 1:** Characteristics of participants (*n* = 26)

**Young people (*n* = 12)**	
Age (years)	
Mean (s.d.)	20.42 (1.73)
Range	18–24
Gender, *n* (%)	
Male	3 (25)
Female	9 (75)
Generation, *n* (%)	
First	2 (16.7)
Second	10 (83.3)
Location, *n* (%)	
London	9 (75)
Leicester	3 (25)
Mental health problem, *n* (%)	
Anxiety	5 (41.7)
Stress	4 (33.3)
Obsessive–compulsive disorder	1 (8.3)
OCPD	1 (8.3)
Depression	1 (8.3)
Current education status, *n* (%)	
Undergraduate	8 (66.7)
Postgraduate	3 (25)
Not in education	1 (8.3)
Sought mental health support, *n* (%)	
Yes	8 (66.7)
No	4 (33.3)

**Parents (*n* = 7)**	
Age (years)	
Mean (s.d.)	47.43 (4.47)
Range	43–56
Gender, *n* (%)	
Male	2 (28.6)
Female	5 (71.4)
Location, *n* (%)	
London	5 (71.4)
Leicester	2 (28.6)
Employment status, *n* (%)	
Employed	2 (28.6)
Unemployed	5 (71.4)

**Community workers (*n* = 7)**	
Age (years)	
Mean (s.d.)	47.57 (8.04)
Range	39–63
Gender, *n* (%)	
Male	5 (71.4)
Female	2 (28.6)
Location, *n* (%)	
London	5 (71.4)
Leicester	2 (28.6)
Job description, *n* (%)	
Psychotherapist/counsellor	2 (28.6)
Centre manager (voluntary organisation)	3 (42.9)
Staff (voluntary organisation)	2 (28.6)
Language (dialects), *n* (%)	
Sylheti-speaking	12 (46.15)
Non-Sylheti-speaking	14 (53.85)

### Thematic framework analysis

**Fig. 1:**
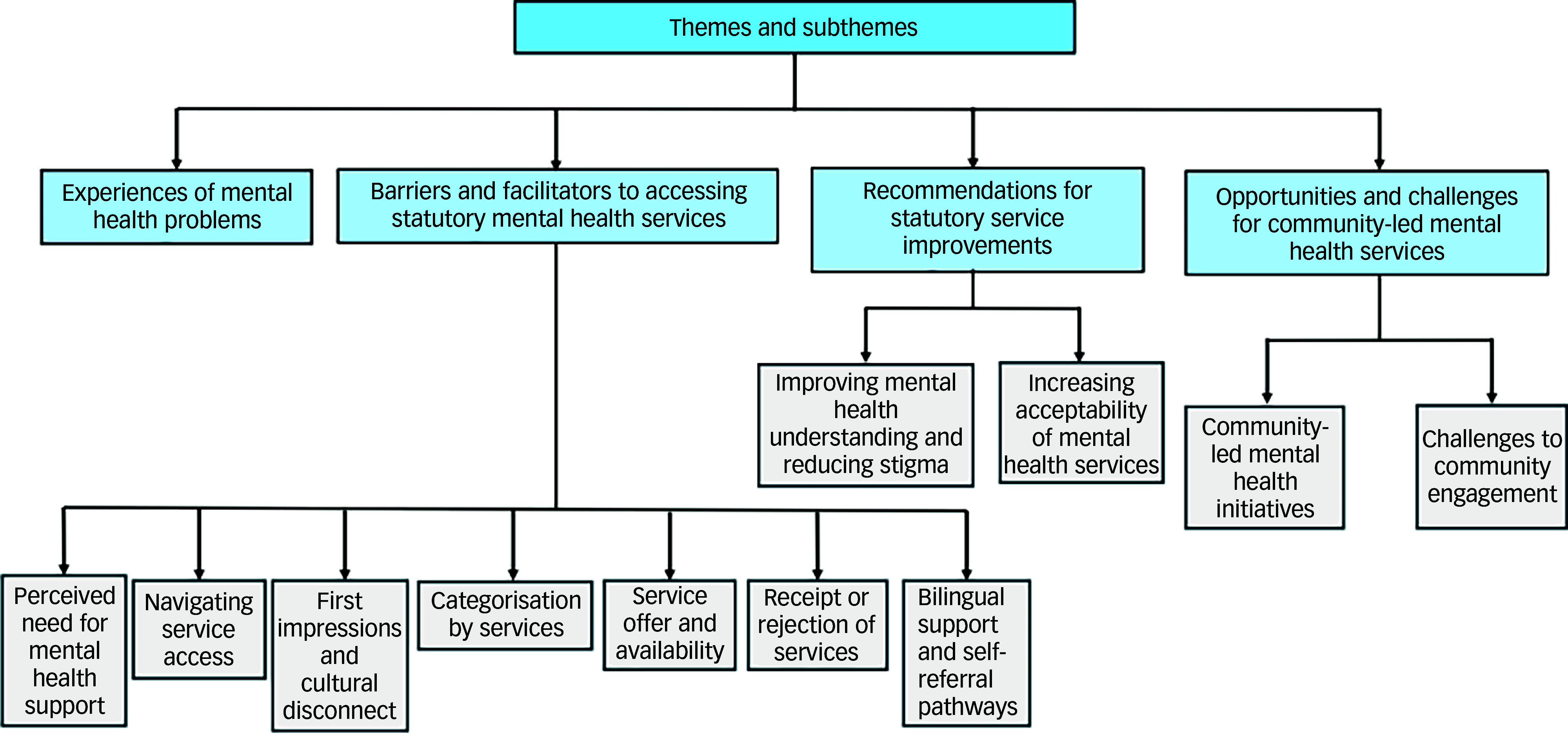
Overview of themes and subthemes identified from qualitative interviews.

Thematic analysis identified four key themes: (a) experiences of mental health problems, (b) barriers and facilitators to accessing statutory mental health services; (c) recommendations for statutory service improvements and (d) opportunities and challenges for community-led mental health services (Fig. 1).

#### Theme 1: Experiences of mental health problems

Young people described feeling overwhelmed, agitated, worried, drained and, at times, low, on edge, anxious or depressed. These emotional struggles, often past and present, disrupted their daily routines, relationships and academic performance. Several mentioned feeling tired and expressed distress through culturally shaped experiences like lack of sleep, drowsiness, poor appetite, feeling achy, being lazy and feeling lonely.

*‘It literally feels like you cannot get out the bed and anything you do won’t change anything, so you feel powerless…very uncertain of what’s going to happen, and you feel very weak…’* (Young person, male, 21 years old)

Parents echoed these concerns, observing their child’s withdrawal and academic disengagement:

*‘It [mental health issue] is significantly affecting [my son’s] life; he can’t focus on anything. He wants to hide from everything; becoming isolated and avoiding social activities or gatherings.’* (Parent, 48 years old)

Mental health challenges were influenced by factors across multiple ecological levels. At the individual level, excessive social media use, gaming and mobile device dependence were reported as contributors to anxiety, depression and social isolation. Parents and community workers viewed these behaviours as detrimental, particularly when they led to unrealistic social comparisons and misinformation.

*‘The younger generation spends more time on TikTok, mobile games, or other social media platforms, where much of the content is fake or filled with rumours…This affects us deeply when we mistake it for reality or compare our lives to it.’* (Parent, 46 years old)

Community workers also pointed to broader relational tensions, including conflicts between parents and within extended family networks, as key stressors affecting youth mental health:

*‘Family conflict has contributed to their anxiety stage. Sometimes it’s their internal family conflicts, sometimes the extended family conflicts…’* (Community worker, 45 years old)

The school environment posed additional challenges. Four participants described intense competition with peers over academic achievement and career prospects, compounded by fear of failure and high parental expectations. Bullying, sometimes from within the same ethnic community, contributed to anxiety, low self-esteem and educational disengagement.

*‘[My daughter] once shared with me that she was bullied in school…When she started university, she saw those same children there, so she didn’t attend university regularly.’* (Parent, 44 years old)

At the macrosystem level, young people described experiences of racism, Islamophobia and cultural misunderstanding in educational and workplace settings.

*‘When I was having my mental health struggles and I used to take a lot of time praying and stuff, my head of year actually ended up getting me in trouble…like they thought I was like a terrorist.’* (Young person, female, 20 years old)

Some also described feelings of inadequacy when adjusting to new academic environments:

*‘For me, it was exactly like impostor syndrome, where I felt like I didn’t have the skills to be there, like in my seminars. I wasn’t contributing or taking my education seriously. Not because I didn’t want to, but I felt like I couldn’t.’* (Young person, female, 21 years old)

#### Theme 2: Barriers and facilitators to accessing statutory mental health services

##### Perceived need for mental health support

Participants described how feelings of shame, fear of judgement, stigma and limited understanding of mental health prevented early help-seeking across generations. Mental health struggles were often hidden or dismissed, with terms like *‘pagol‘* (*‘*crazy*‘*) and *‘matha kharap‘* (*‘*mad*‘*) used casually, reinforcing shame and misunderstanding:

*“They [community members] just call them ‘crazy’… Like, there’s no awareness of what actually mental health is.”* (Young person, male, 21 years old)

Young men described pressures to conform to masculine stereotypes and how vulnerability is seen as a weakness.

*‘There is that sort of stigma still around Bangladeshi boys*…*as a man, you kind of proceed as needing to be tough.’* (Young person, male, 19 years old)

All participant groups highlighted a profound lack of mental health knowledge, leading to problems being overlooked or misinterpreted.

*‘*…*They [parents] don’t understand much. They think, “Oh, you’re upset, everyone gets upset once in a while. It’s nothing out of the ordinary.’* (Young person, female, 18 years old)

Although younger generations have begun to develop a language around mental health, confusion persists about differentiating normal stress from clinical conditions. A community worker noted that terms like anxiety, depression and feeling low are commonly used by young people to describe their challenges.

##### Navigating service access

For families who recognised a young person’s mental health needs, navigating support was often filled with barriers. One key challenge was caregivers’ limited English proficiency, which restricted their ability to advocate for their children, communicate with providers and access information. One parent explained that they heavily relied on their children to navigate systems at their NHS general practice, but the children’s reluctance to help made accessing care even more difficult. Also, discomfort and feelings of personal shame further hindered the youth’s ability to engage with the statutory mental health system.

*‘People [Bangladeshi] are hesitant to seek support from the NHS…taboo, feeling of shame, ‘I can deal with myself’, ‘what’s the benefit of talking to people about it’.’* (Community worker, 39 years old)

A lack of awareness about available mental health services also created difficulties, leaving individuals unsure of how or where to seek support.

##### First impressions and cultural disconnect

Initial encounters with mental health professionals played a crucial role in shaping attitudes toward continued engagement. Many participants felt that therapists lacked cultural competence, leading to discomfort during sessions.

*‘My sister took therapy from an English lady and my sister was not happy as the therapist didn’t understand our cultural things.’* (Community worker, 41 years old)

Furthermore, community workers mentioned that a lack of mental health professionals with Bangladeshi heritage in boroughs such as Southwark, Camden and Islington further contributed to dissatisfaction, as people struggle to find culturally or religiously aligned therapists.

##### Categorisation by services

Participants expressed dissatisfaction with general practitioners (GPs) misattributing emotional distress to physical problems or offering general advice, such as recommending rest or exercise, without addressing mental health issues. One young person explained,

*‘With my GP I had issues because when I initially told them how I’m feeling, they were like, “Oh, maybe you’re anaemic.” Then they sent me for a blood test. But I wasn’t anaemic at all.’* (Young person, female, 24 years old)

Similarly, several young people felt therapists unfamiliar with Bangladeshi culture misunderstood or pathologised their family dynamics.

*‘I felt like I had to explain myself a lot…like…my family’s not bad…like, I think a White British person might say, “Oh, is that not a bit abusive?” So, I have to tiptoe around issues and not explain them to the full extent because I’m worried about them thinking badly of my family.’* (Young person, female, 20 years old)

##### Service offer and availability

Community workers pointed to budget cuts, staff shortages and limited service provision as barriers to effective long-term mental health support. Short-term therapy was also seen by one young person as insufficient for addressing complex mental health concerns.

*‘I ended up having one session. Although [the therapist was] very nice, it felt like they don’t really know how to provide proper counselling—because they give you five sessions and then you’re on your way…they sort of messed up!’* (Young person, female, 20 years old)

Long waiting lists further discouraged help-seeking.

*‘Sometimes [the GP] refers to a psychiatrist or psychologist, but there’s a long queue. And there’s a saying, “by the time I get my appointment, my mental health will have already improved!”’* (Community worker, 45 years old)

##### Receipt or rejection of services

Participants mentioned that families often seek religious or supernatural remedies over medical treatment for their mental health problems and prefer Muslim therapists to ensure their faith and world view were understood and respected.

*‘…you know, when you have those [mental health] problems, they [traditional Bengalis] say things like Jinn [evil spirit] or like, “oh, she’s possessed…she needs Ruqyah [Islamic healing method]”, things like that, like exorcisms.’* (Young person, female, 20 years old)

There was also fear and mistrust of conventional treatments, with young people noting that many in the community were wary of therapy and antidepressants, fearing dependency on medication.

Despite challenges, participants acknowledged some strengths within the statutory mental health system:

##### Bilingual support and self-referral pathways

NHS providers, particularly GPs, were praised for providing multilingual information and interpreter access, facilitating clearer communication. In addition, the NHS Talking Therapies self-referral system was appreciated for its simplicity and prompt response, allowing individuals to initiate help-seeking without a GP referral.

*‘The government, local authorities, voluntary sectors, and the NHS have many centres and services available in multiple languages like English, Bengali, Hindi and Somali.’* (Community worker, 52 years old)

#### Theme 3: Recommendations for statutory service improvements

##### Improving mental health understanding and reducing stigma

Participants emphasised the need for educational programmes to improve community understanding of mental health issues, reduce stigma, and normalise open conversations:

*‘…you need to create awareness among the people so that they can understand what is happening in their family, what a mental health issue is, and what the symptoms of mental health issues are.’* (Community worker, 45 years old)

To improve awareness, participants recommended culturally relevant outreach strategies, such as using ethnic media, mosques and community centres, as well as incorporating storytelling, art therapy and role-play at fairs and cultural events.

*‘People watch ethnic channels like Channel S, NTV [UK-based Bangla language TV channels]. So media can be a powerful tool…Some people like to read newspapers. So we can use both print and electronic media, mosque, leafletting, and word of mouth.’* (Community worker, 45 years old)

Participants also suggested integrating mental health education into school curricula, focusing on resilience, emotional regulation and coping strategies. One community worker highlighted that many young people struggle with understanding their emotions and forming a sense of identity, underlining the need for such support.

##### Increasing acceptability of mental health services

Participants stressed the need for culturally sensitive language, relatable examples and bilingual or multilingual support groups to enhance accessibility.

*‘It’s easier to talk to someone who is from the same background. You might even speak the same language.’* (Young person, female, 21 years old)

Young people strongly preferred therapists who share or deeply understand their cultural background and gender, as they perceived it could help with communication and building trust.

*‘I would go to a Muslim female professional, maybe South Asian or another ethnic minority, because they have that ingrained knowledge of where I’m coming from.’* (Young person, female, 21 years old)

Community workers advocated for a multidimensional approach, incorporating self-referral pathways with support from a trusted contact person.

*‘I don’t think young people will come to the community centre and say, “I don’t feel right…I feel anxious.” It’s when they go to a service or youth club, where they have a point of contact, that they might open up and share. Then, a professional or contact person can pick it up or take the next steps and approach services gradually.’* (Community worker, 41 years old)

Participants also highlighted the importance of flexible therapy options, including both online and in-person sessions, and adjusting session frequency or length to suit individual preferences. Another key concern was the need for early intervention and quick access to support, avoiding long waiting periods.

*‘It is better to recognise this as quickly as possible, if somebody’s suffering from mental problems, they need support without wasting time because earlier intervention can help them to get better.’* (Community worker, 63 years old)

Finally, nine participants emphasised the importance of placing services in accessible and familiar locations, such as schools and universities:

*‘…like you’re in a hostile environment at home, and when you go to school, it’s a safe environment where you can possibly share. Having that place is very important.’* (Young person, male, 19 years old)

### Theme 4: Opportunities and challenges for community-led mental health services

#### Community-led mental health initiatives

Participants strongly advocated for family-focused mental health initiatives, emphasising the need to educate parents and older generations about conditions such as anxiety and depression. They believed that increasing parental awareness and motivation was critical for ensuring that children receive appropriate support at home. Parents also expressed a willingness to engage in mental health programmes, especially if they perceived these as being tailored for their children’s benefit. Young people highlighted the importance of structured community spaces to promote open discussions and a sense of belonging.

*‘Maybe creating a community hub having a drop-in or like a space where young people can come in and get the right support.’* (Young person, female, 22 years old)

Peer support was seen as valuable. Youth and community workers highlighted the potential peer-led initiatives to create relatable, safe spaces for mental health conversation:

*‘Peers will be the best way. Young people will be trained and then deliver it, and they will feel so relatable*.*’* (Community worker, 48 years old)

Participants identified trusted community figures, such as leaders, role models and religious authorities, as crucial in bridging the gap between statutory services and the community, reducing stigma and promoting openness.

*‘It might be good to have a Bengali speaker just showing how they’ve gone through, like being not so well mentally and then reaching out to support, and how it’s improved them…’* (Young person, male, 19 years old)

Participants also called for better coordination between GPs, hospitals and community organisations to provide integrated and holistic care. Bilingual and culturally competent professionals, especially those with a Bangladeshi background, were seen as vital for delivering services in ways that resonate with community needs.

#### Challenges to community engagement

A key barrier to community-based support was fear of stigma and concerns about confidentiality. Participants described hesitancy to seek help from within their own community, worried that personal information might be shared or misused.

*‘…if you want support from the community, the barrier will be finding the right person. In my experience in the Bengali community, people like to talk about other people… if I share something, will she keep it a secret, or will she tell others, and then people might make fun of me?’* (Young person, female, 24 years old)

Additionally, insufficient funding, resources and staffing can create significant obstacles for community organisations.

*‘We can train organisational staff or members of the community to deliver these services. But the main issue would be the funding to deliver these kinds of services.’* (Community worker, 52 years old)

## Discussion

The study investigated experiences of mental health problems and priorities and preferences for mental health support among British Bangladeshi youth through interviews with young people, parents and community workers. Using thematic analysis informed by key theoretical models, four themes were identified regarding mental health issues, barriers and facilitators to accessing services, recommendations for service improvement and opportunities for strengthening community-led support.

The mental health challenges reported by our participants build on prior research indicating that internalising problems are evident among Bangladeshi and other ethnic minority youth in the UK.^28^ Participants also described culturally specific expressions of distress, including supernatural explanations like Jinn possession^13,29^ and the tendency to somatise symptoms, particularly where open discussions of mental health are discouraged.^30^ In many contexts, emotional distress is seen as situational rather than medical, which can delay diagnosis and treatment.^31,32^ These cultural frameworks significantly influence how mental health is experienced and when support is sought.

British Bangladeshi youth identified family conflict as a key contributor to mental distress. Intergenerational tensions and differing cultural expectations between parents and children often led to isolation and emotional strain, consistent with acculturation theory and associated stress.^33,34^ Research shows that such conflicts hinder communication and support within Bangladeshi families.^13,35^ Although British Bangladeshis navigate both heritage and Western cultural norms,^36^ traditional values often persist, shaping mental health perceptions and influencing help-seeking behaviours.^37^ Young people also stressed social media overuse, bullying and discrimination (e.g. racism, Islamophobia) as major stressors, reflecting a broader tendency within Bangladeshi communities to view mental distress as socially rooted rather than biologically based.^2,31,32^ This situational framing is common across South Asian cultures.^38^ Consistent with minority stress theory,^39^ participants linked discrimination in schools and communities to psychological distress and imposter syndrome. Structural racism has similarly been shown to exacerbate mental health issues among ethnic minorities.^5,40^

Stigma, shame and fear of judgement were key barriers to help-seeking in the Bangladeshi community, echoing findings that internalised stigma and cultural taboos, driven by fears of gossip and social exclusion, discourage early intervention.^5,31^ Community and family judgement, often branding individuals as *‘*crazy*’* or *‘*weak*’*, intensifies this reluctance.^13^ Cultural ideals of masculinity, which prize endurance and self-reliance, further prevent men from seeking support.^13,41^ Although young people in the Bangladeshi community are increasingly informed about mental health through school lessons, social media and peer conversations, their understanding may be limited. For example, research suggests young people in the UK have difficulty distinguishing between everyday stress and clinical conditions.^42^ Foulkes^42^ argues that although increasing awareness about mental health has many benefits, it may result in young people mislabelling normal developmental struggles (like sadness or stress) as mental health disorders, particularly when appropriate support systems are lacking. Language barriers meant that parents in this study struggled to access mental health services on behalf of their children and found it difficult to communicate with providers, relying on assistance from English-speaking family members.^5,31^ A lack of awareness about services further limited access.^3^

Cultural and systemic barriers within statutory mental health services, such as child and adolescent mental health services and GP practices, shaped participants’ experiences with services, echoing prior findings.^13,40^ A lack of cultural competence and underrepresentation of diverse therapists led to feelings of misunderstanding and detachment, especially among Sylheti speakers (a dialect of Bangla), who often view mental health through non-Western lenses.^5^ Additionally, GPs frequently misattributed mental health issues to physical symptoms, delaying diagnosis and eroding trust, reflecting known patterns of somatisation in the Bangladeshi community.^5,43^

Systemic issues like staff shortages, funding cuts, and long waiting lists continue to limit access to timely, effective care, reinforcing previous findings on how under-resourced services discourage early help-seeking and exacerbate mental health crises.^3,5,43^ Traditional beliefs and negative perceptions about pharmacological treatments, particularly among older Bangladeshis, often frame mental illness as spiritual (e.g., Jinn possession), leading individuals to seek help from Imams rather than professionals, limiting service uptake and reinforcing stigma.^2,5,13^

Despite barriers, participants acknowledged strengths in current statutory services, such as bilingual support, access to interpreters, culturally diverse professionals in certain boroughs and the user-friendly NHS Talking Therapies referral system. Although cultural competence is crucial for effective care,^44^ these provisions may be limited to areas with large Bangladeshi populations, raising concerns about unequal access elsewhere.

The study identified culturally sensitive strategies to improve access, including mosque-based outreach, Bengali media campaigns and creative methods like art therapy to reduce stigma and promote early engagement. Key recommendations from participants included expanding bilingual, culturally aligned services and flexible therapy options (online and in-person) to address wait times, language barriers, and service acceptability, echoing earlier findings.^5,31^ The findings also highlighted that community-led initiatives have the potential to address generational gaps in awareness. Educating parents can enhance their capacity to better understand and respond to their children’s mental health needs. Community spaces like peer groups and drop-in centres may additionally provide culturally safe avenues for dialogue and connection. Involving young leaders, community workers and religious figures, such as Imams, can also reduce stigma and promote help-seeking, especially when mental health is framed in ways that align with cultural and religious values.^5,13,31^

However, a key challenge is the fear of judgement and breaches of confidentiality when disclosing mental health concerns within close social networks, discouraging individuals from seeking informal or community-based support. Past findings also emphasise the need for neutral, external spaces to ensure privacy.^44^ Limited funding and resources also constrain community organisations’ capacity to provide scalable services, reflecting inequalities in resource allocation. These findings indicate the need for community-based, culturally sensitive mental health support tailored to Bangladeshi communities, helping to improve engagement, accessibility and mental health outcomes. Representatives from these communities should be actively involved in the development of future programmes and policies, ensuring that support is both relevant and sustainable.

### Strengths and limitations

This study included diverse perspectives from young people, parents and community workers, providing a more nuanced understanding of mental health needs across generations. This triangulation of views revealed important areas of convergence, such as shared concerns about stigma and access barriers, as well as divergence, particularly in how young people and parents conceptualised mental health and their preferred support mechanisms. Collaboration with community organisations, bilingual materials and interviews in participants’ preferred languages ensured inclusivity. The study captured perspectives from both London and Leicester, although samples were not systematically compared with each other because of differences in size and context.

However, the study has some limitations. Despite efforts to include a wide variety of participants, time constraints and limited staff capacity meant that only three out of 14 organisations agreed to collaborate, limiting the diversity of organisational insights. Although formal screening was not conducted (as described in the Method), this approach may have led to the inclusion of participants whose mental health conditions did not meet diagnostic criteria, which could affect the specificity of the findings. Furthermore, the study did not conduct member checking to validate participants’ accounts, which may have affected the credibility of the findings. Relying solely on interviews is another limitation; incorporating additional qualitative methods such as focus groups or ethnographic research could have helped triangulate and strengthen the results.

In conclusion, this study highlights how cultural stigma, intergenerational tensions and systemic barriers within statutory services shape British Bangladeshi youths’ experiences of mental health. Sustainable improvement requires enhancing cultural competence, reducing access inequalities and strengthening trusted, community-based and bilingual support. Embedding community partnerships and youth participation within service design can ensure that mental health support is inclusive, contextually relevant and effective.

## Supporting information

Shahnaz et al. supplementary material 1 Shahnaz et al. supplementary material

Shahnaz et al. supplementary material 2 Shahnaz et al. supplementary material

## Data Availability

The data that support the findings of this study are available from the corresponding author, I.S., upon reasonable request.

## Supplementary material

The supplementary material is available online at https://doi.org/10.1192/bjo.2026.10999

## References

[1] Office for National Statistics. Ethnic Group, England and Wales: Census 2021. Office for National Statistics, 2022 (https://www.ons.gov.uk/).

[2] Birmingham City Council. Bangladeshi Community Health Profile. Birmingham City Council, 2021 (https://www.birmingham.gov.uk/publichealth).

[3] Haywood C , Mac an Ghaill M. Young Bangladeshi Peoples Experience of Transition to Adulthood. Joseph Rowntree Foundation, 2005 (https://archive.org/details/youngbangladeshi0000macg).

[4] Bamford J , Klabbers G , Curran E , Rosato M , Leavey G. Social capital and mental health among black and minority ethnic groups in the UK. J Immigr Minor Health 2021; 23: 502–10. 32623610 10.1007/s10903-020-01043-0 PMC8068661

[5] Bisby N , Singam S , Beattie A , Ekiko F , Clilverd A. The Mental Health Needs of the Bangladeshi Community in Camden: An Action Research Project . Camden and Islington Mental Health and Social Care Trust, 2003 (https://bwhp.co.uk/pdf/reports/mental_health_action_research_report.pdf).

[6] Association of London Government (ALG). Sick of Being Excluded: Improving the Health and Care of London’s Black and Minority Ethnic Communities. ALG, 2000 (https://journals.sagepub.com/doi/abs/10.1177/003803850203600401).

[7] Sabella K , Davis M , Munson MR. The transition to adulthood: a critical developmental period within a changing social-contextual landscape. In Handbook of Research on Emotional and Behavioral Disorders (eds TK Curby , ML Wehby , JR Lloyd ): 16–31. Routledge, 2020.

[8] Blanco C , Okuda M , Wright C , Hasin DS , Grant BF , Liu SM , et al. Mental health of college students and their non-college-attending peers: results from the National Epidemiologic Study on Alcohol and Related Conditions. Arch Gen Psychiatry 2008; 65: 1429–37. 19047530 10.1001/archpsyc.65.12.1429 PMC2734947

[9] Kessler RC , Berglund P , Demler O , Jin R , Merikangas KR , Walters EE. Lifetime prevalence and age-of-onset distributions of DSM-IV disorders in the National Comorbidity Survey Replication. Arch Gen Psychiatry 2005; 62: 593–602. 15939837 10.1001/archpsyc.62.6.593

[10] Patel V , Flisher AJ , Hetrick S , McGorry P. Mental health of young people: a global public-health challenge. Lancet 2007; 369: 1302–13. 17434406 10.1016/S0140-6736(07)60368-7

[11] National Health Service (NHS) Digital. Psychological Therapies: Annual Report on the Use of IAPT Services, England 2019–20. NHS Digital, 2020 (https://www.digital.nhs.uk/pubs/psycther1920).

[12] Mind. Mental Health Crisis Care: Commissioning Excellence for Black and Minority Ethnic Groups: A Briefing For Clinical Commissioning Groups. Mind, 2013 (https://www.mind.org.uk/media-a/4371/bme-commissioning-excellence-briefing.pdf).

[13] Alam S. British-Bangladeshi Muslim men: removing barriers to mental health support and effectively supporting our community. Cogn Behav Ther 2023; 16: 38.

[14] MacDonald H , Martin M , Clark E , Dehn Lunn A , Gkiouleka A , Harasgama S , et al. Evidence Brief: What Works – Community Engagement and Empowerment to Address Health Inequalities. Health Equity Evidence Centre, 2024 (https://www.heec.co.uk/resource/what-works-community-engagement-and-empowerment-to-address-health-inequalities/#ref9). 10.1016/j.puhip.2026.100773 PMC13053805 41952881

[15] Bangladeshi Mental Health Forum. About Us . Bangladeshi Mental Health Forum, 2025 (https://bangladeshimentalhealth.org/).

[16] Health and Adult Social Care Scrutiny Committee. Addressing Health and Wellbeing Inequalities Among the Bangladeshi Community in Camden: Update on the Work of the 2015 ‘Improving Outcomes for the Bangladeshi Community in Camden’ Scrutiny Group, and Current Initiatives. London Borough of Camden, 2024 (https://democracy.camden.gov.uk/ieListDocuments.aspx?CId=634&MID=10587#AI63076).

[17] Roshni Ghar. About Us . Roshni Ghar, 2025 (https://www.roshnighar.org.uk/).

[18] Poole L , Rickford R , Martinez A , Gill P , Frith H , Ronaldson A , et al. Mental health among British South Asians: reflecting on granularity. Br J Psychiatry 2025; 227: 637–8. 40685921 10.1192/bjp.2025.117

[19] Tong A , Sainsbury P , Craig J. Consolidated criteria for reporting qualitative research (COREQ): a 32-item checklist for interviews and focus groups. Int J Qual Health Care 2007; 19: 349–57. 17872937 10.1093/intqhc/mzm042

[20] Malterud K , Siersma VD , Guassora AD. Sample size in qualitative interview studies: guided by information power. Qual Health Res 2016; 26: 1753–60. 26613970 10.1177/1049732315617444

[21] Guest G , Namey E , Chen M. A simple method to assess and report thematic saturation in qualitative research. PLoS ONE 2020; 15: e0232076. 32369511 10.1371/journal.pone.0232076 PMC7200005

[22] Gale NK , Heath G , Cameron E , Rashid S , Redwood S. Using the framework method for the analysis of qualitative data in multi-disciplinary health research. BMC Med Res Methodol 2013; 13: 117. 24047204 10.1186/1471-2288-13-117 PMC3848812

[23] Ritchie J , Lewis J. Qualitative Research Practice: A Guide for Social Science Students and Researchers. SAGE, 2003.

[24] Bronfenbrenner U. The Ecology of Human Development: Experiments by Nature and Design. Harvard University Press, 1979.

[25] Guy-Evans O. Bronfenbrenner’s Ecological Systems Theory. Simply Psychology, 2024 (https://www.simplypsychology.org/bronfenbrenner.html).

[26] Gask L , Bower P , Lamb J , Burroughs H , Chew-Graham C , Edwards S , et al. Improving access to psychosocial interventions for common mental health problems in the United Kingdom: narrative review and development of a conceptual model for complex interventions. BMC Health Serv Res 2012; 12: 249. 22889290 10.1186/1472-6963-12-249 PMC3515797

[27] Brown JSL , Lisk S , Carter B , Stevelink SAM , Van Lieshout R , Michelson D. How can we actually change help-seeking behaviour for mental health problems among the general public? Development of the ‘PLACES’ model. Int J Environ Res Public Health 2022; 19: 2831. 35270523 10.3390/ijerph19052831 PMC8909998

[28] Bains S , Gutman LM. Mental health in ethnic minority populations in the UK: developmental trajectories from early childhood to mid adolescence. J Youth Adolesc 2021; 50: 2151–65. 34436736 10.1007/s10964-021-01481-5 PMC8505297

[29] Dein S , Alexander M , Napier AD. Jinn, psychiatry and contested notions of misfortune among east London Bangladeshis. Transcult Psychiatry 2008; 45: 31–55. 18344251 10.1177/1363461507087997

[30] Bhui K , Bhugra D. Mental illness in Black and Asian ethnic minorities: pathways to care and outcomes. Adv Psychiatr Treat 2002; 8: 6–33.

[31] Rodie S , Park L , Chowdhury F. Study of a Needs Assessment of the Bangladeshi and Wider Muslim Community: Summary Report. Suffolk and North East Essex Integrated Care System, 2022 (https://www.sneeics.org.uk/wp-content/uploads/2023/02/BWAE-needs-assessment-summary-report.pdf).

[32] Karasz A. Cultural differences in conceptual models of depression. Soc Sci Med 2005; 60: 1625–35. 15652693 10.1016/j.socscimed.2004.08.011

[33] Bhui K , Stansfeld S , Head J , Haines M , Hillier S , Taylor S , et al. Cultural identity, acculturation, and mental health among adolescents in East London’s multiethnic community. J Epidemiol Community Health 2005; 59: 296–302. 15767383 10.1136/jech.2003.014456 PMC1733051

[34] Berry J. Immigration, acculturation, adaptation. Appl Psychol 1997; 46: 5–68.

[35] Kapasi R. No More Small Portions of Services Please: A Report of the Issues and Needs Highlighted by the Mental Health Training for Bangladeshi Workers. Bengali Women’s Health Project and Camden & Islington Community Health Services, 2001 (https://www.bwhp.co.uk/pdf/reports/mental_health_training.pdf).

[36] Helman CG. Culture, Health and Illness: An Introduction for Health Professions. Wright, 1990.

[37] Sheikh S , Furnham A. A cross-cultural study of mental health beliefs and attitudes towards seeking professional help. Soc Psychiatry Psychiatr Epidemiol 2000; 35: 326–34. 11016528 10.1007/s001270050246

[38] Desjarlais R , Eisenberg L , Good B , Kleinman A. World Mental Health: Problems and Priorities in Low Income Countries. Oxford University Press, 1995.

[39] Meyer IH. Prejudice, social stress, and mental health in lesbian, gay, and bisexual populations: conceptual issues and research evidence. Psychol Bull 2003; 129: 674–97. 12956539 10.1037/0033-2909.129.5.674 PMC2072932

[40] Nazroo J. Ethnicity and Mental Health: Findings from a National Community Survey. Policy Studies Institute, 1997 (https://books.google.com/books/about/Ethnicity_and_Mental_Health.html?id=YY3sAAAAMAAJ).

[41] Clement S , Schauman O , Graham T , Maggioni F , Evans-Lacko S , Bezborodovs N , et al. What is the impact of mental health-related stigma on help-seeking? A systematic review of quantitative and qualitative studies. Psychol Med 2015; 45: 11–27. 24569086 10.1017/S0033291714000129

[42] Foulkes L. The problem with mental health awareness. Br J Psychiatry 2024; 225: 337–8. 39399916 10.1192/bjp.2024.106

[43] Bhui K , Bhugra D (eds). Ethnicity: An Agenda for Mental Health. Gaskell, 1999 (https://pmc.ncbi.nlm.nih.gov/articles/PMC1118760/).

[44] Bhui K , Warfa N , Edonya P , McKenzie K , Bhugra D. Cultural competence in mental health care: a review of model evaluations. BMC Health Serv Res 2007; 7: 15. 17266765 10.1186/1472-6963-7-15 PMC1800843

