# Supplementary material for: Mental health support for British Bangladeshi youth: multi-stakeholder qualitative study of priorities and preferences
Source: BJPsych Open. 2026 Mar 10;12(2):e86. doi: 10.1192/bjo.2026.10999 (PMC13107320; doi:10.1192/bjo.2026.10999)
Supplement: Shahnaz et al. supplementary material 2 — Shahnaz et al. supplementary material [file S2056472426109995sup002.docx]

**Semi-structured interview guide – Young people**

Introduction:

- Introduction of self
- Introduction of study
- Key points
  - length of interview (45-60 minutes)
  - no right or wrong answers - exploring perspectives
  - participation is voluntary - right to withdraw participation
  - confidentiality/anonymity
  - how findings will be reported
  - interview will be recorded
  - Questions? Happy to proceed?

| **Theme** | **Question** | **Probe** |
| --- | --- | --- |
| Ice breaking | Could you please, tell me about yourself? | background, family, education, |
| Mental Health | I want to learn more about the mental health of young people with Bangladeshi backgrounds. I wondered if you could start by telling me what mental health means to you. | What is good mental health? What is poor mental health? |
|  | I would like to know about your mental health. How would you describe it? | Now, in the past, what happened that influenced your mental health?  What terms do you use to describe it? |
|  | Some people find it easy to talk about their mental health whereas others find it difficult. What is your experience of talking about your mental health? | Probe for experiences discussing with family, friends, health workers  With whom/where/when do you feel most comfortable/uncomfortable? Why? |
|  | I now want to ask you about young people in general, especially those with a Bangladeshi background like you…  In your opinion, what do young people need for good mental health? What leads to poor mental health? | positive and negative factors |
|  | What does it feel like to have poor mental health? | Any local term/word  How does it affect day to day?  What might people do differently from when they have good mental health?  What might they think or do?  How might they behave? |
| Community resources | What helps young people to feel better when they have mental health problem?  What are the main people or places for young people who need support for their mental health? | Any local, internet, phone-based support  Friends, family, school/college?  What would stop/help young people to access these supports? |
|  | Now I want you to think about the kind of help that might be available in the community. Can I start by asking what you understand by the word community?  What is your community? | the first thing that comes to your mind, any personal experiences of community activity/involvement  neighbourhood, online, educational institute, work |
|  | Who in your local community do you trust and feel connected to, and why? | Experience of getting support or guidance from these individuals or organizations |
|  | In what way might these community members help with your mental health and mental health of other young people like you? | Personal experience, Impact (positive/negative), |
| Mental Health Stigma | Now, shifting our focus, I would like to explore your thoughts on a different aspect. Can I ask what is your perceptions of the attitudes within your local community toward mental health issues?  Considering the diverse perspectives within communities, I'd also like to know your thoughts as an ethnic minority member. How would you describe the attitudes within your local community toward mental health issues from this perspective? | Evidence of any stigma, discrimination  impact on young people |
|  | Can you share any experiences of discrimination or stigma when accessing services that you believe are unique to your cultural background or ethnic identity? | In school/community, impact on your mental health and your willingness to seek mental health support |
|  | How do you think are your experiences of stigma in relation to accessing support for mental health different to those encountered by other ethnic minority groups? | any particular cultural or linguistic barriers you've encountered that may have affected your access to mental health resources compared to other ethnic minority groups?  any common misconceptions or stereotypes related to mental health within your ethnic community that might impact seeking help? |
|  | How do you think your community could tackle stigma related to being from an ethnic minority group? | Personal experience, preferences |
| Community-based Services | How do you believe mental health support be improved to better meet the needs of young Bangladeshi individuals? | the benefits and concerns of such a program, thought/preference for referral system  considering their cultural, and linguistic backgrounds? |
|  | In some places young people have benefitted from participating in talking therapy/learning problem solving strategies where they share problems, feelings and thoughts with a therapist and sometimes as part of a group. They might have one or more sessions every week for 6 or 8 weeks. What do you think about this? Would you be interested in taking part in something like this if you had a mental health problem? | What do you like about the idea? What are your concerns about the idea? |
|  | How would you like to get the help? | getting help in a group or being offered individually, mix the age/gender in a same session |
|  | What additional support do you, as a young person, wish to receive from your community regarding mental health? | Any specific mental health services or activities you believe your community could provide |
|  | What possible barriers could stop you from reaching this kind of support? | stigma, parents won’t allow/embarrassed/shy talking about problems, distance, how they are treated by friends/family/community  minority discrimination or prejudice |

**Thank and finish**

- anything else they would like to add

**Semi-structured Interview guide- Community members/Representatives from the community or voluntary organisation**

Introduction:

- Introduction of self
- Introduction of study
- Key points
  - length of interview (45-60 minutes)
  - no right or wrong answers - exploring perspectives
  - participation is voluntary - right to withdraw participation
  - confidentiality/anonymity
  - how findings will be reported
  - interview will be recorded
  - Questions? Happy to proceed?

| **Theme** | **Questions** | **Probe** |
| --- | --- | --- |
| Ice breaking | Could you please, tell me about yourself? | Background, education, profession |
| Youth’s Mental Health Problems | I want to learn more about the mental health of young people. I wondered if you could start by telling me what mental health means to you. | the first thing that comes to your mind, What is good mental health? What is poor mental health?  importance of the mental health of students/youth to you |
|  | Since you are involved in supporting young people, can you share the most common problems you have experienced in students/youth who belong to an ethnic minority group? | problems related to education, relationship, behavioural activities |
|  | In your view, what things might affect the mental health of students or young people who belong to an ethnic minority group? | factors related to discrimination, prejudice, stigma, culture, linguistic, family, school, community, peers, and person-related |
|  | How do these things affect their mental health? | depression and anxiety symptoms, behaviour/body language/activities /mood/ relationships |
| Community-based Services | Now, let's turn our attention to the specific context of the Bangladeshi community in the UK. I'm interested in hearing your insights on how mental health is currently perceived or discussed within this community. How do you believe the topic of mental health is currently perceived or discussed within the Bangladeshi community in the UK? | Any misconceptions, specific things about the culture or how people interact in the Bangladeshi community in the UK that affect how they talk about and understand mental health |
|  | Considering the insights you've shared, what do you think could be done or provided in this community to support mental health of young people better? | Any existing service or resources, culture/language sensitive support |
|  | In your opinion, who can support better these young individuals regarding their mental health issues? | School teachers, parents, peers, community members, representatives from voluntary organisations, local leaders |
|  | According to you, what referral system would work better with the young people? | Teacher/parent/self-referral |
|  | What do you think are the potential concerns and challenges about having mental health services in community? | anticipated stigma, challenges in community members' involvement, and others |
|  | What ideas or strategies would you recommend addressing these concerns? | Any examples of successful strategies in other ethnic minority communities that you think could be adapted to address these concerns here |

**Thank and finish**

- anything else they would like to add

**Semi-structured interview guide- Family Members**

Introduction:

- Introduction of self
- Introduction of study
- Key points
  - length of interview (45-60 minutes)
  - no right or wrong answers - exploring perspectives
  - participation is voluntary - right to withdraw participation
  - confidentiality/anonymity
  - how findings will be reported
  - interview will be recorded
  - Questions? Happy to proceed?

| **Theme** | **Question** | **Probe** |
| --- | --- | --- |
| Ice breaking | Could you please, tell me about yourself? | Background, education, work, number of children |
| Mental Health | I want to learn more about the mental health of young people. I wondered if you could start by telling me what the word ‘mental health’ means to you? | Understanding the difference between mentally healthy and having poor mental health |
|  | Since you have a young person in your family, what do you think are the most common mental health problems in young people? | tension, stress, sadness, depression, anxiety or fearfulness and low confidence, concern over the way they look, exam stress, anger issues, destructive/disruptive behaviours, bullying, attention problems, learning problems, suicidal thoughts, substance abuse, drugs, etc |
|  | In your view, what things might affect the mental health of young people? | factors related to discrimination, prejudice, stigma, culture, linguistic, family, school, community, peers, and person-related |
|  | How do you think these problems affect the lives of young people? | managing at school, at home and with friends |
|  | How do young persons usually handle these kinds of issues? | Activities/behaviour to manage emotions and stress, use of alcohol, drugs, and violent outbursts |
| Community-based services | Now, shifting our focus to support networks, I'd like to hear your thoughts. In your opinion, who do you think can provide better support for young individuals dealing with mental health issues? | School teachers, parents, peers, community members, representatives from voluntary organisations, local leaders |
|  | How do community organisations/leaders currently help with young people’s mental health? | Existing resources, Any culture sensitive support, specific service for their community |
|  | What difficulties or obstacles do you think exist in the support provided by community organizations or leaders for young people's mental health? | Belong to an ethnic minority group |
|  | How do you think the way people see a person from your groups while seeking mental health support is different from how it might be for others from different ethnic backgrounds? | any particular cultural or linguistic barriers that may have affected the access to mental health resources compared to other ethnic minority groups?  any common misconceptions or stereotypes related to mental health within your ethnic community that might impact seeking help? |
|  | I'm curious to know how different approaches to supporting young people from Bangladeshi communities with their mental health have worked in your opinion. What kinds of programs or support do you think might be helpful for them? | Counselling/talking therapies/problem focused therapy, challenges and concerns, and strategies to address these challenges |
|  | How do you think such a program would help the young people? | Anticipated outcomes, anticipated benefits to youth |
|  | What is your view of involving family members in such programs? | As a referrer, other suggestions |

**Thank and finish**

- anything else they would like to add
